# Retraction: Deep learning in public health: Comparative predictive models for COVID-19 case forecasting

**DOI:** 10.1371/journal.pone.0321232

**Published:** 2025-04-09

**Authors:** The PLOS One Editors

The *PLOS One* Editors retract this article due to concerns about peer review integrity, authorship, and adherence to the journal’s publication requirements on reporting and data availability. Among other concerns, most of Table 1 and Figs 2–10 were published previously in [2–4], which was not cited or discussed in [1]. We regret that the issues were not addressed prior to the article’s publication.

All authors did not agree with the retraction.

## References

[1] Tariq MU, Ismail SB. Deep learning in public health: Comparative predictive models for COVID-19 case forecasting. PLoS ONE. 2024;19(3): e0294289. doi: 10.1371/journal.pone.0294289 38483948 PMC10939212

[2] Tariq MU, Ismail SB, Babar M, Ahmad A. Harnessing the power of AI: Advanced deep learning models optimization for accurate SARS-CoV-2 forecasting. PLoS ONE. 2023;18(7):e0287755. doi: 10.1371/journal.pone.0287755 37471397 PMC10359009

[3] Tariq MU, Ismail SB, Babar M, Ahmad A. Correction: Harnessing the power of AI: Advanced deep learning models optimization for accurate SARS-CoV-2 forecasting. PLoS ONE. 2023;18(12): e0296111. doi: 10.1371/journal.pone.0296111 38096185 PMC10721022

[4] The PLOS One Editors. Retraction: Harnessing the power of AI: Advanced deep learning models optimization for accurate SARS-CoV-2 forecasting. PLoS ONE. 2025;20(4): e0321233. doi: 10.1371journal.pone.0321233 10.1371/journal.pone.0321233 PMC11981156 40203015

